# The Mechanism of Speech Processing in Congenital Amusia: Evidence from Mandarin Speakers

**DOI:** 10.1371/journal.pone.0030374

**Published:** 2012-02-08

**Authors:** Fang Liu, Cunmei Jiang, William Forde Thompson, Yi Xu, Yufang Yang, Lauren Stewart

**Affiliations:** 1 Center for the Study of Language and Information, Stanford University, Stanford, California, United States of America; 2 Music College, Shanghai Normal University, Shanghai, China; 3 Department of Psychology, Macquarie University, Sydney, Australia; 4 Department of Speech, Hearing and Phonetic Sciences, University College London, London, United Kingdom; 5 Institute of Psychology, Chinese Academy of Sciences, Beijing, China; 6 Department of Psychology, Goldsmiths, University of London, London, United Kingdom; University of Barcelona, Spain

## Abstract

Congenital amusia is a neuro-developmental disorder of pitch perception that causes severe problems with music processing but only subtle difficulties in speech processing. This study investigated speech processing in a group of Mandarin speakers with congenital amusia. Thirteen Mandarin amusics and thirteen matched controls participated in a set of tone and intonation perception tasks and two pitch threshold tasks. Compared with controls, amusics showed impaired performance on word discrimination in natural speech and their gliding tone analogs. They also performed worse than controls on discriminating gliding tone sequences derived from statements and questions, and showed elevated thresholds for pitch change detection and pitch direction discrimination. However, they performed as well as controls on word identification, and on statement-question identification and discrimination in natural speech. Overall, tasks that involved multiple acoustic cues to communicative meaning were not impacted by amusia. Only when the tasks relied mainly on pitch sensitivity did amusics show impaired performance compared to controls. These findings help explain why amusia only affects speech processing in subtle ways. Further studies on a larger sample of Mandarin amusics and on amusics of other language backgrounds are needed to consolidate these results.

## Introduction

As a neuro-developmental disorder of music processing, congenital amusia (amusia hereafter) provides a unique opportunity for studying the cognitive and neural mechanisms underlying language and music processing [1]. This is because despite suffering from severe musical impairments in everyday life [2]–[3], individuals with amusia (amusics hereafter) only demonstrate subtle problems with linguistic tone and intonation processing under laboratory conditions [4]–[8]. The apparent domain specificity of amusia (severely impaired musical processing and largely spared linguistic processing) has been explained by the ‘Melodic Contour Deafness Hypothesis’, according to which amusics have pitch direction discrimination deficit for both speech and music, although this deficit has a more significant impact on music processing than linguistic intonation processing [1: 233].

A number of factors may account for the robustness of intonation perception in amusia. First, in a non-tonal language like English, the acoustic realization of focus (e.g., ‘**John** loves Mary’ versus ‘John loves **Mary**’, with focus on the first and last word, respectively) is not only dependent on variation in pitch direction, but also on the large pitch movement of the focused word and the lowered pitch of the following words [9], which can explain amusics' normal performance on focus identification and discrimination [1]–[2], [8], [10]. Second, when pitch direction plays a significant role in signifying statements and questions in English [11], amusics can detect these differences as long as the pitch contrasts exceed their pitch direction discrimination thresholds and when there are other cues (syntactic, semantic, and contextual) in the signal to aid understanding [8]. Thus, spared linguistic but impaired musical abilities in amusics may arise because linguistically meaningful pitch contrasts in non-tonal languages are relatively large compared to the pitch intervals used in music [1], [12]–[13]. Indeed, when exposed to relatively small pitch direction contrasts in the final words of statements and questions in English and French, most amusics showed impaired performance on discrimination, identification, and imitation of these utterances [4], [6].

It remains unclear whether the ‘Melodic Contour Deafness Hypothesis’ holds for speakers of tone languages in which pitch distinguishes meaning at the lexical level. For example, ‘ma’ in Mandarin signifies different meanings depending on different lexical tones [14]–[15]: ‘mother’ (Tone 1, High), ‘hemp’ (Tone 2, Rising), ‘horse’ (Tone 3, Low), and ‘to scold’ (Tone 4, Falling). Mandarin tones are primarily characterized by the height and shape of fundamental frequency (F_0_) contours, though other acoustic cues such as duration, intensity, and phonation type (e.g., creaky voice) also play a role [16]–[19]. Although such features suggest a strong connection between tone languages and music, recent findings have confirmed that tone language speakers also suffer from amusia, and the prevalence of this disorder is similar (around 4%) for speakers of tone and non-tonal languages [5], [7], [20]–[21].

Like English/French amusics [4], [6], [8], Mandarin-speaking amusics exhibit subtle problems with intonation processing when exposed to small pitch differences in statement- and question-final syllables [5]. However, in linguistic tone processing, a subgroup of Mandarin amusics performed at ceiling on discrimination of lexical tones carried by the same segment, e.g., ‘yu2’ versus ‘yu3’ (Tones 2 and 3 on the same segment ‘yu’), but showed impaired discrimination on tones carried by different segments, e.g., ‘shan1’ versus ‘wu4’ (Tones 1 and 4 on different segments ‘shan’ and ‘wu’) [7]. Furthermore, despite demonstrating normal lexical tone production, these amusics were unable to identify the tones by names (such as 1/2/3/4 in ‘hua1/hua2/hua3/hua4’) as well as controls [7].

Given these mixed results, the authors of [7] proposed two possible sources of deficits for the ‘lexical tone agnosia’ in their subgroup of Mandarin amusics: 1) ‘impaired pitch tracking system’, and 2) ‘low executive or attentional control’ [7: 2641]. However, the low-level ‘pitch tracking’ deficit hypothesis seems unlikely given that, a) the pitch excursion sizes of the tones used in [7] were rather large (2–17 semitones on average, which likely exceeds amusics' pitch discrimination thresholds; [6], [22]–[23]), and, b) amusics with ‘lexical tone agnosia’ showed normal performance on discrimination of tones that shared the same segments (demonstrating normal ‘pitch tracking’ abilities). In contrast, the ‘low executive or attentional control’ hypothesis seems plausible: around 40% of amusics may have attention deficits [24] and amusia is associated with deficits in phonemic/pitch awareness [25]–[27]. More precisely, it is possible that ‘lexical tone agnosia’ reflects impaired phonological awareness, i.e., awareness of the sound structure of a word [28]–[29]. In fact, previous studies have indicated that even normal Mandarin speakers (including children and adults) have difficulty identifying lexical tones using tone names (Tone 1/2/3/4) and discriminating tone pairs when segments are also varied [30]–[32]. This is likely because task difficulty and linguistic complexity interfere with phonological awareness [33]. Therefore, it remains an open question whether Mandarin-speaking amusics have pitch-processing deficits for lexical tones in their native language (rather than due to lack of phonological awareness).

The current investigation examined the mechanism of speech processing in congenital amusia in Mandarin speakers from the following four perspectives. First, assuming that amusia is a domain-general pitch-processing deficit as proposed by the ‘Melodic Contour Deafness Hypothesis’ [1] and demonstrated by several recent studies [5]–[6], [8], we expect Mandarin amusics to show tone processing deficits in speech when the tonal contrasts are relatively small (not greatly exceeding their pitch discrimination thresholds) and when the tones are carried by the same segments (not involving high demand on attentional/executive control or phonological awareness). Therefore, we took a different approach than [7] in which labeling was required for tone identification and attentional/executive control was essential for tone discrimination due to the use of different segments. Instead, we designed the tone perception tasks as identification and discrimination of Mandarin words that shared the same segments but had small tonal contrasts (1.5–4.1 semitones on average; Table S1; the words were represented by corresponding Chinese characters, in order to reduce the demand for phonological awareness). We hypothesized that Mandarin amusics' pitch-processing deficit would be revealed in the language domain under such conditions.

Second, given that amusics rarely report language problems in daily life [5]–[6], it was necessary to examine how and why they are able to manage speech communication with such a severe pitch-processing deficit. Therefore, in contrast to the design in [5] where short statements and questions were manipulated to differ primarily in the pitch pattern of the final syllable, we conducted intonation perception tasks that required participants to identify and discriminate naturally-spoken statements and questions that differed in various acoustic characteristics (F_0_, duration, and intensity) across the entire utterances. It was predicted that Mandarin amusics would be able to perform as well as controls on these tasks owing to the additional non-pitch-based cues (duration and intensity).

Third, it is unclear how stimulus type (speech versus non-speech analogs) affects pitch processing in amusia. Some studies suggest that amusics are better able to process natural speech than tone analogs [2], [5], [10], while others have failed to observe this difference [6], [8]. To examine further the effect of stimulus type on pitch processing in amusia, we employed gliding tone analogs of the tone and intonation stimuli in the above two tasks to compare amusics' performance on speech versus non-speech materials.

Finally, to explore the link between pitch processing in low-level psychophysical tasks and high-level linguistic tasks, the current study also included two pitch threshold tasks that used adaptive-tracking forced-choice procedures to determine participants' thresholds for detection of pitch change and discrimination of pitch direction, as in [6].

## Materials and Methods

### Participants

Participants were recruited through advertisements in the bulletin board system of universities in Beijing. Volunteers were first screened by author CJ through a phone interview inquiring about their musical (dis)abilities. Depending on whether they reported difficulty carrying a tune and detecting an out-of-tune note in a melody, these volunteers were classified as either potential amusics or possible controls. Suitable volunteers were then invited to the lab for diagnosis of amusia using the Montreal Battery of Evaluation of Amusia (MBEA) [34]. Consisting of six subtests (each 30 trials, scored using number of correct responses out of 30), the MBEA assesses individuals' abilities to discriminate pitch changes in melodies in three pitch-related subtests (contour, interval, and scale), and measures their musical aptitudes for rhythm, meter, and memory in the other three subtests. To separate amusics from controls, participants' pitch composite scores (the sum of the scores on the three pitch subtests) were calculated, and those scored at or below 65 were confirmed as amusics [6], [34]. In the end, thirteen amusics and thirteen matched controls agreed to participate in the study. All were undergraduate or Master's students at Beijing universities with Mandarin Chinese as their native language and having no formal extra-curricular musical training (see Table S2 for details). None of the participants reported speech/hearing impairments or neurological/psychiatric disorders. Table 1 summarizes the characteristics of the two groups. While controls showed significantly better performance than amusics on all MBEA subtests, the two groups were comparable in sex, handedness, age, and education (in years).

**Table 1:** Characteristics of the amusic (*n* = 13) and control (*n* = 13) groups.

Group	Sex	Handedness	Age	Education	Scale	Contour	Interval	Rhythm	Meter	Memory	Pitch composite
Amusic											
Mean	8F	2L	24.08	16.62	16.92	19.31	18.69	21.92	19.54	21.54	54.92
SD	5M	11R	2.93	2.53	3.33	2.90	2.98	4.54	4.03	4.48	6.97
Control											
Mean	9F	0L	24.69	17.92	27.00	26.85	26.38	27.08	26.31	28.23	80.23
SD	4M	13R	1.84	0.95	1.91	1.72	1.61	1.71	2.32	2.01	3.59
*t*-test											
*t*			0.64	1.74	9.46	8.06	8.18	3.83	5.24	4.91	11.64
*p*			0.53	0.09	<0.0001	<0.0001	<0.0001	<0.0001	<0.0001	<0.0001	<0.0001

F = female; M = male; L = left; R = right; scores on the six MBEA subtests are in number of correct responses out of 30; the pitch composite score is the sum of the scale, contour, and interval scores; *t* is the statistic of the Welch two sample *t*-test (two-tailed, *df* = 24).

### Materials

The speech stimuli used in the word and intonation tasks were recorded by a 20-year-old female student at Goldsmiths, University of London, who was born and raised in Beijing until the age of 18, with Beijing Mandarin as her native language. The recording was done in a soundproof booth using Praat [35], with 44.1 kHz sampling rate and 16-bit amplitude resolution.

### Word stimuli

Thirty-three word pairs were used in the word identification/discrimination tasks. Among them, there were eight monosyllabic pairs (e.g., 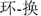, huan2-huan4, ‘hoop’-‘change’), ten disyllabic pairs (e.g., 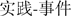, shi2jian4-shi4jian4, ‘practice’-‘event’), seven 3-syllable pairs (e.g., 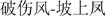, po4shang1feng1-po1shang4feng4, ‘tetanus’-‘a phoenix on the hill’), and eight 4-syllable pairs (e.g., 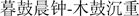, mu4gu3chen2zhong1-mu4gu3chen2zhong4, ‘evening drums and morning bells’-‘wooden drums are heavy’). The two words in each pair shared the same segments but differed in tonal composition. The frequencies of usage of the words in mono- and di-syllabic pairs were closely matched (paired *t*-test: *t*(14) = 0.06, *p* = 0.95, with 4 words having missing frequencies, 2 in the same pair and 2 in different pairs) [36]. Given that 3- and 4-syllable words are rare in Chinese [36], it was not always possible to find pairs of words with the same segments and matched frequencies but different tones. Therefore, compounds or phrases were used in some 3- and 4-syllable word pairs. The nature of the words (words versus pseudo-words), however, did not affect participants' performance, as shown in the Results section.

Previous research indicates that focused words have significantly larger pitch excursion sizes than non-focused words and that pitch ranges of post-focus words are compressed and lowered compared to pre-focus words in Mandarin [37]. In order to solicit word stimuli that have relatively small pitch movements but with different sizes, the speaker was instructed to produce the sixty-six words under both pre- and post-focus conditions within the same context (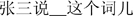 [‘ZhangSan said the word __’]). In the pre-focus condition, the target words occurred before the focus of the carrier sentence (the final word 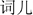), whereas in the post-focus condition, the same set of target words occurred after the focus of the carrier sentence (the initial word 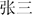). These target words were later extracted from their sentential contexts, resulting in thirty-three word pairs in each focus condition as test stimuli. The absence of tonal contexts has either negative [38] or no effect [31] on tone identification in Mandarin. Neither effect is likely to have significant consequences for the results of the current study, since both amusic and control groups were exposed to the same set of context-free stimuli. On the other hand, not including sentential contexts might have helped to prevent ceiling performance in the two groups.

In order for the two words in each pair to differ primarily in pitch, one was selected (randomly) as the base (e.g., po4shang1feng1), and the other as the pitch template (e.g., po1shang4feng4). Using a custom-written Praat script, the pitch template was first adjusted to match the base in duration, syllable-by-syllable (the duration adjustment had no significant influence on the F_0_ profile of the word, since the two words in each pair had closely matched durations. Paired *t*-test: *t*(65) = 0.998, *p* = 0.32). The pitch of the base was then replaced by that of the pitch template. This created a new stimulus with the segment(s) of the base but pitch contour of the pitch template. The original bases and their new counterparts then served as test stimuli for word discrimination/identification. In total, 66 word pairs (33 in each focus condition) were created following this procedure.

**Figure 1:**
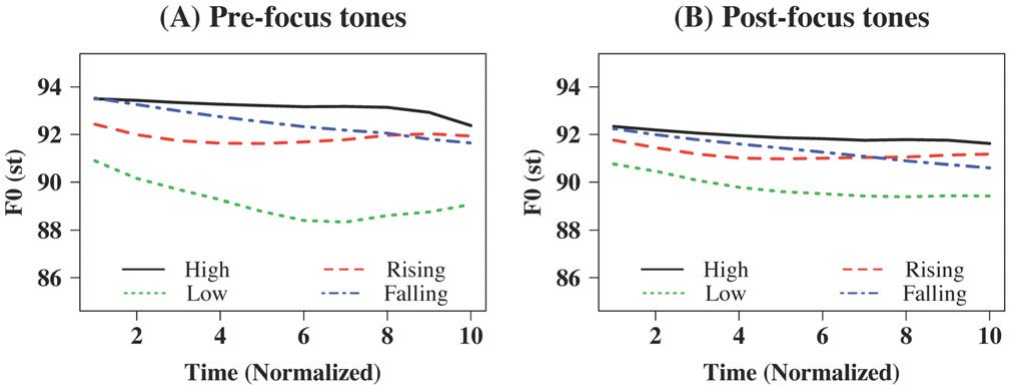
Mean time-normalized F_0_ contours (in semitones, or st; st = 12 * log_2_(Hz), Hz = 2^(st/12)^) of the four Mandarin tones. (A) in pre-focus words, and (B) in post-focus words. The F_0_ contours of the High tone (Tone 1) were averaged across 47 tokens, those of the Rising tone (Tone 2) 51 tokens, those of the Low tone (Tone 3) 9 tokens, and those of the Falling tone (Tone 4) 55 tokens.

Within the 33 word pairs in each focus condition, there were 162 individual tones, among which 47 were High (Tone 1), 51 Rising (Tone 2), 9 Low (Tone 3), and 55 Falling (Tone 4). The scarcity of the Low tone in the stimuli was deliberate because this tone is often characterized by phonation type (i.e., creaky voice) rather than F_0_ [16]. Figure 1 shows mean time-normalized F_0_ contours (in st) of the four Mandarin tones, averaged across all the syllables that shared the same tones in the stimulus sets under pre- versus post-focus conditions. Table 2 displays acoustic characteristics of these tones in post- versus pre-focus words, with those under the post-focus condition (except for Tone 3) having significantly lower mean F_0_ and shorter duration than those under the pre-focus condition. However, the two sets of tones did not differ significantly in pitch excursion size, which ranged between 1.5 and 4.1 st on average across different tones, or in glide rate/time (see Table S1 for detailed definitions and measurements). Furthermore, pre- and post-focus words exhibited similar pitch ranges across the tone(s) within the word [maximum F_0_ – minimum F_0_; post-focus mean (SD): 3.48 st (1.45), pre-focus: 3.67 st (2.03), *t* (65) = −0.68, *p* = 0.50)]. In order to examine whether words under different focus conditions were processed differently, pre- and post-focus words were tested separately in different blocks.

**Table 2:** Acoustic characteristics of the tones in post- versus pre-focus words.

Tone	Acoustic characteristics	Post-focus	Pre-focus	Paired *t*-test (two-tailed)
Tone 1 (*n* = 47)	Mean F_0_ (st)	91.97 (1.16)	93.23 (0.81)	*t(46)* = −5.91, *p*<0.0001
	Duration (s)	0.11 (0.02)	0.12 (0.03)	*t(46)* = −4.52, *p*<0.0001
	Mean intensity (dB)	83.91 (1.77)	83.42 (2.27)	*t(46)* = 1.52, *p* = 0.13
Tone 2 (*n* = 51)	Mean F_0_ (st)	91.25 (1.39)	91.95 (1.09)	*t(50)* = −3.77, *p* = 0.0004
	Duration (s)	0.11 (0.03)	0.12 (0.04)	*t(50)* = −4.02, *p* = 0.0002
	Mean intensity (dB)	82.97 (2.36)	83.10 (2.42)	*t(50)* = −0.35, *p* = 0.73
Tone 3 (*n* = 9)	Mean F_0_ (st)	89.90 (0.65)	89.62 (1.63)	*t(8)* = 0.70, *p* = 0.51
	Duration (s)	0.10 (0.02)	0.10 (0.04)	*t(8)* = −0.97, *p* = 0.36
	Mean intensity (dB)	83.18 (2.42)	82.74 (3.24)	*t(8)* = 0.58, *p* = 0.58
Tone 4 (*n* = 55)	Mean F_0_ (st)	91.48 (1.06)	92.66 (0.89)	*t(54)* = −7.09, *p*<0.0001
	Duration (s)	0.11 (0.03)	0.12 (0.04)	*t(54)* = −3.51, *p* = 0.0009
	Mean intensity (dB)	83.01 (1.89)	82.70 (2.11)	*t(54)* = 0.99, *p* = 0.33

Data are means (SD). Mean F_0_ (in semitones, or st) is the average fundamental frequency of the tone; duration (in seconds, or s) is the length of the tone; mean intensity (in decibels, or dB) is the mean-energy intensity of the tone; Tone 1 = High; Tone 2 = Rising; Tone 3 = Low; Tone 4 = Falling.

### Intonation stimuli

**Figure 2:**
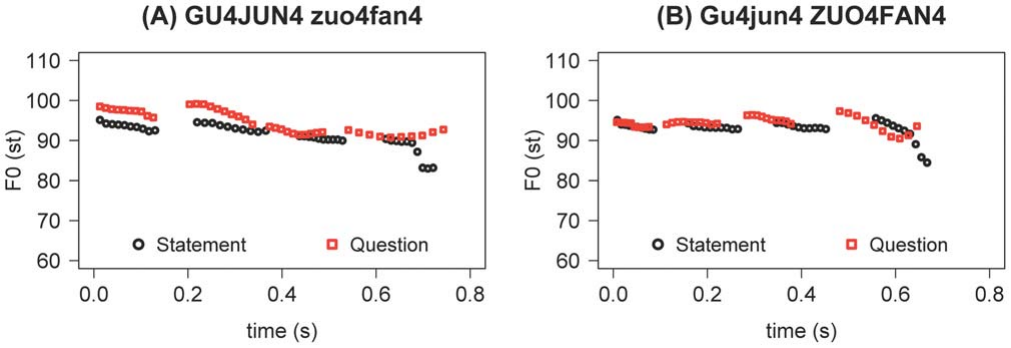
Real-time (in s) F_0_ contours (in st) of two statement-question pairs. (A) the first two syllables ‘Gu4Jun4’ were focused, and (B) the last two syllables ‘zuo4fan4’ were focused. The Chinese characters of the sentences are ‘
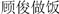
’ [‘GuJun cooks the rice’], in which all the syllables carried the Falling tone (Tone 4).

Intonation stimuli comprised 20 statement-question pairs that shared the same word sequence but differed in intonation. These utterances ranged from 3 to 7 syllables and consisted of only High/Falling tones. They were naturally spoken with either an initial or a final focus. Figure 2 shows real-time F_0_ contours of two pairs of statements and questions, with those in the left panel containing an initial focus and those on the right having a final focus (see Table S3 for how these sentences were formed). As can be seen, the significant differences between these statements and questions not only lie in F_0_ (questions showing overall higher pitches than statements), but also in their duration patterns, with statement-final syllables showing significantly shorter durations than the corresponding question-final syllables (0.11 s versus 0.22 s in Figure 2A and 0.12 s versus 0.18 s in Figure 2B). Acoustic characteristics of the 40 statements and questions and their final syllables are summarized in Table 3. Paired *t*-tests indicate that statements had significantly lower mean F_0_, lower mean intensity, and wider pitch range than questions both as a whole and on the final syllable. Furthermore, statement-final syllables had significantly shorter duration and smaller glide rate than question-final syllables.

**Table 3:** Acoustic characteristics of the statements and questions and their final syllables.

Sentence type		Sentence				Final syllable					
		Mean F_0_	Duration	Mean Intensity	Pitch Range	Mean F_0_	Duration	Mean Intensity	Glide Size	Glide Time	Glide Rate
Statement (*n* = 20)	*μ*	92.88	0.86	78.98	11.35	91.20	0.13	75.90	6.82	0.10	−61.19
	*σ*	2.17	0.22	3.44	7.27	4.61	0.04	6.89	7.20	0.05	84.70
Question (*n* = 20)	*μ*	93.66	0.89	80.09	7.75	92.50	0.19	77.68	3.59	0.12	−22.78
	*σ*	1.37	0.22	2.42	3.94	2.45	0.04	5.06	4.38	0.04	37.15
*t*-test	*t*	−2.22	−1.70	−2.86	2.46	−1.80	−5.89	−2.50	2.54	−1.65	−2.41
	*p*	0.04	0.11	0.01	0.02	0.09	<0.0001	0.02	0.02	0.12	0.03

Mean F_0_ (in st) is the average fundamental frequency of the sentence (or the final syllable); duration (in s) is the length of the sentence (or the final syllable); mean intensity (in dB) is the mean-energy intensity of the sentence (or the final syllable); pitch range (in st) is defined as the difference in fundamental frequency between maximum and minimum F_0_ of the sentence; glide size (in st) is the pitch excursion size of the final syllable ( = maximum F_0_−minimum F_0_); glide time (in s) is the duration between maximum and minimum F_0_ of the final syllable; glide rate (in st/s) = glide size/glide time; *μ* = mean; *σ* = standard deviation; *t* is the statistic of the paired *t*-test (two-tailed, *df* = 19).

### Gliding tone analogs of word and intonation stimuli

Using the technique described in [6], [8], [10], [39], gliding tone analogs of the word/intonation stimuli were created with Praat. These tone analogs had the same pitch and rhythmic patterns as the original stimuli, but were made of complex tones that consisted of the F_0_ plus seven odd harmonics of the syllable(s) in the stimuli, leading to a clarinet-like sound quality. Examples of the speech stimuli and their tone analogs can be found at http://www.phon.ucl.ac.uk/home/yi/SoundExamples2/SoundExamples.html. To achieve roughly equal loudness, the amplitudes of all stimuli were normalized by increasing the peak value to the maximum using Praat.

### Procedure

Experiments were conducted in a quiet room at the Institute of Psychology, Chinese Academy of Sciences in Beijing, China. Written informed consent forms were obtained from all participants before testing. The protocol was reviewed and approved by the Goldsmiths, University of London Ethics Committee. The entire testing session (with regular breaks) took about two hours on average, during which the participants completed six word perception, three intonation perception, and two pitch threshold tasks for the present study, and a number of listening/singing tasks for another study.

### Word discrimination and identification

The word perception tasks were presented to all participants in separate blocks in the same order: 1) pre-focus word discrimination (discrimination of the word pairs in pre-focus condition), 2) pre-focus glide discrimination (discrimination of the gliding tone analogs of the word pairs in pre-focus condition), 3) pre-focus word identification (identification of the words in pre-focus condition), 4) post-focus word discrimination (discrimination of the word pairs in post-focus condition), 5) post-focus glide discrimination (discrimination of the gliding tone analogs of the word pairs in post-focus condition), and 6) post-focus word identification (identification of the words in post-focus condition). Two other tasks were interspersed in between these word tasks, separating them with roughly 10-minute intervals.

In the discrimination tasks, each of the 33 stimulus pairs appeared in both ‘same’ (randomly selected 16 pairs as word 1 – word 1 and the other 17 pairs as word 2 – word 2) and ‘different’ configuration (randomly selected 16 pairs as word 1 – word 2 and the other 17 pairs as word 2 – word 1). Thus, there were 66 stimulus pairs (33 ‘same’ pairs and 33 ‘different’ pairs) in the discrimination tasks and 66 individual stimuli in the identification tasks. All discrimination/identification stimuli were pseudo-randomized and presented to the participants in the same order, with 750 ms interstimulus interval (in the discrimination tasks) and 1500 ms intertrial interval.

Four practice trials (with different stimuli than the experimental trials) were given before each of the first three tasks to familiarize the participants with the experimental procedure and materials. During testing, participants were required to judge as quickly and accurately as possible whether the two words/glides were the same or different in the discrimination tasks, and which word they had heard in the identification tasks (by choosing the corresponding Chinese characters of the words). Responses were recorded with key presses combined with reaction times. The Chinese characters of ‘same’ [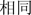] and ‘different’ [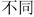] (for the discrimination tasks) and those of the word pairs (for the identification tasks) were displayed on the computer screen (one to the left and one to the right) to indicate to the participants which key to press (‘q’ for the left and ‘p’ for the right). The experimental protocols were the same across word/glide discrimination tasks and pre-/post-focus conditions. Participants were not informed that the stimuli were related.

### Statement-question discrimination and identification

The three intonation perception tasks were also presented to the participants separately in fixed order: 1) statement-question discrimination (discrimination of the statement-question pairs), 2) gliding tones discrimination (discrimination of the gliding tone analogs of the statement-question pairs), and 3) statement-question identification (identification of the statements and questions). Two other tasks were again administered in between these intonation tasks, separating them with 10–15 minute gaps.

Four practice trials (with different stimuli than the experimental trials) were given before each task. There were 40 individual stimuli or stimulus pairs in each task. These stimuli were arranged and presented to the participants in the same way as the word perception tasks. Participants were asked to respond via a key press (‘q’ or ‘p’) whether the two sentences or tone sequences were the ‘same’ [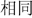] or ‘different’ [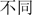] in the discrimination tasks and whether they heard a ‘statement’ [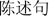] or ‘question’ [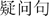] in the identification task, while their reaction times were recorded.

### Pitch threshold tasks

As in [6], participants' thresholds for pitch change detection and pitch direction discrimination were evaluated with adaptive-tracking procedures using a 3-interval, 2-alternative forced-choice oddball (‘odd-one-out’) design. In the pitch change detection task, participants were required to report which of the three pure tones (two steady-state and one gliding, each 600 ms in duration, with 600 ms interstimulus interval) contained a glide, thus detecting a pitch change. In the pitch direction discrimination task, participants were asked to report which of the three gliding tones differed in direction (rising versus falling) from the other two, thus discriminating the direction of pitch change. The threshold (in semitones) was calculated as the mean pitch excursion size of the target glide in the last six reversals using the ‘2 down, 1 up’ staircase method.

### Scoring and statistical analyses

In keeping with previous studies [2], [6], [8], [10], performance was scored as the percentage of hits minus the percentage of false alarms (%H-%FA) for the discrimination tasks, and as the percentage of correct responses (%Correct) for the identification tasks. Specifically, a hit was achieved when a ‘different’ pair was correctly judged as different, whereas a false alarm arose when a ‘same’ pair was judged as different.

Statistical analyses were conducted using R, ‘a language and environment for statistical computing’ [40]. Data were analyzed using mixed-effects ANOVAs. Results were also confirmed (but not reported here in the interest of space) with non-parametric methods (Wilcoxon rank sum test and Wilcoxon signed rank test), as amusics' scores on three tasks (there were in total 22 tests) did not follow a normal distribution (Shapiro-Wilk normality tests: pre-focus glide discrimination: *W* = 0.75, *p* = 0.002; pre-focus word identification: *W* = 0.84, *p* = 0.02; pitch direction discrimination: *W* = 0.86, *p* = 0.03). Correlations were evaluated with the rank-based measure of association, Kendall's *τ* statistic (two-sided). Generalized linear mixed models were fit using the lme4 package for R to determine the effects of stimulus characteristics on participants' responses, with individual participants and stimulus items as random effects and stimulus characteristics as fixed effects [41]. The analyses of reaction time data are not reported because no group difference was found in regard to this measure (but see Tables S5 and S7 for results).

## Results

### Word discrimination and identification

**Figure 3:**
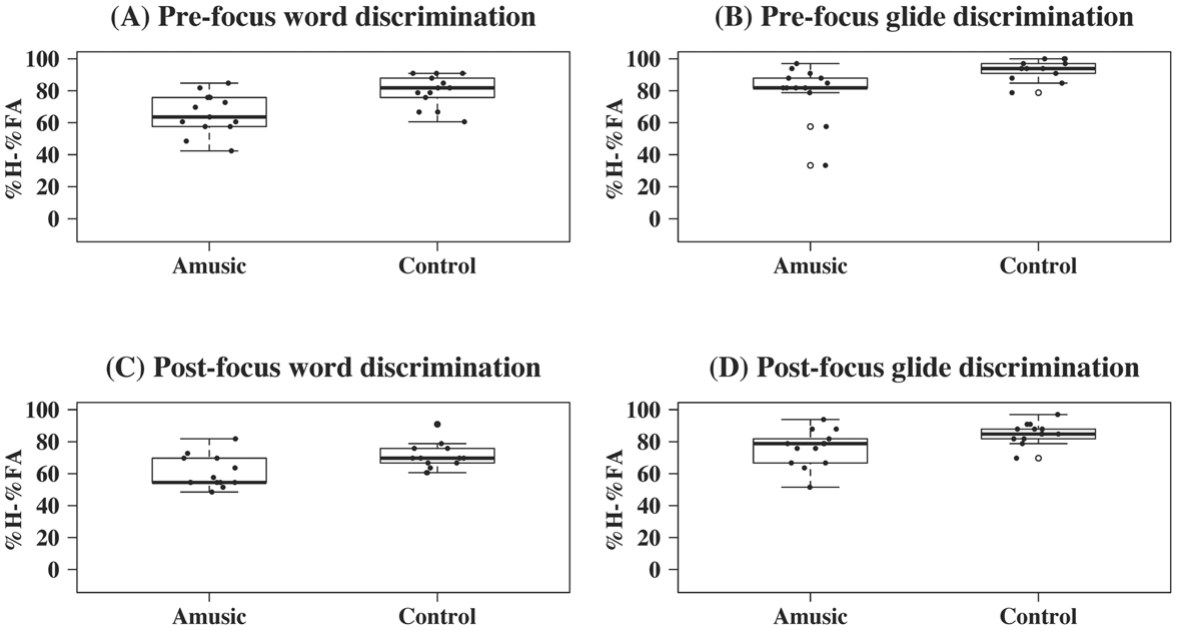
Boxplots of amusics and controls' scores on the four word discrimination tasks (in ‘percentage of hits – percentage of false alarms’; %H – %FA). (A) pre-focus word discrimination, (B) pre-focus glide discrimination, (C) post-focus word discrimination, and (D) post-focus glide discrimination. Individual scores are represented by black dots, with those at the same horizontal level having identical values, and those lying beyond the whiskers being outliers (which are further indicated by open circles in the middle).

Figure 3 shows the results of the word/glide discrimination tasks (see Tables S4 and S5 for individual scores and reaction times). Mixed-effects ANOVA with Subject (individual participants) as the random effect, Group (amusic versus control) the between-subject factor, and Stimulus (word versus glide) and Focus (pre versus post) the within-subject factors revealed significant main effects of Group [F(1,24) = 13.71, *p* = 0.001], Stimulus [F(1,72) = 81.56, *p*<0.0001], and Focus [F(1,72) = 16.37, *p* = 0.0001]. No significant interactions were found. This indicates that, regardless of focus condition, amusics performed significantly worse than controls on both word discrimination and glide discrimination. Both groups performed significantly better on glide discrimination than word discrimination [amusics: F(1,36) = 32.60, *p*<0.0001; controls: F(1,36) = 56.53, *p*<0.0001]. While controls achieved significantly better performance under the pre-focus condition than the post-focus condition [F(1,36) = 20.83, *p*<0.0001], the effect of focus on amusics' performance was only marginally significant [F(1,36) = 2.87, *p* = 0.099].

There was a significant positive correlation between performances on pre-focus word discrimination and pre-focus glide discrimination for amusics (*z* = 2.50, *p* = 0.01, *τ* = 0.55). Amusics' performances on pre- and post-focus word discrimination were also positively correlated (*z* = 2.92, *p* = 0.004, *τ* = 0.65). No other correlations reached statistical significance.

Consistent with previous findings [6], [8], most errors made by amusics in the discrimination tasks were misses rather than false alarms (77.0% versus 23.0% in pre-focus word discrimination; 76.9% versus 23.1% in post-focus word discrimination; 86.0% versus 14.0% in pre-focus glide discrimination; 91.3% versus 8.7% in post-focus glide discrimination). Thus, errors were mainly caused by amusics' insensitivity to the differences between the stimuli. A generalized linear mixed model was fit to examine the effects of stimulus characteristics on amusics' responses to ‘different’ pairs, in which stimulus type (word versus glide), focus condition (pre-focus versus post-focus), stimulus length (1–4 syllables), number of different tones between the two stimuli in a pair (1–4), number of compounds/pseudo-words in a pair (0, 1), and the absolute difference in pitch range between the two stimuli in a pair were included as fixed effects, and individual amusics and stimulus items were treated as random effects. The results on stimulus type and focus condition were consistent with the findings based on the ANOVAs on the whole stimulus sets (‘same’ plus ‘different’ pairs). That is, amusics performed better on glide discrimination than on word discrimination (*z* = 4.69, *p*<0.0001), and they also achieved better performance on pre-focus stimuli than on post-focus stimuli (*z* = 2.40, *p* = 0.02). Furthermore, amusics performed better when the absolute difference in pitch range between the two stimuli in a pair was larger (*z* = 2.13, *p* = 0.03). The other fixed effects (stimulus length, number of different tones between the two stimuli in a pair, and number of compounds/pseudo-words in a pair) did not contribute significantly to amusics' performance on detecting the difference between the word/glide stimuli in ‘different’ pairs. Similar analysis on controls revealed that they also performed better on glide discrimination than on word discrimination (*z* = 7.01, *p*<0.0001), and on pre-focus stimuli than on post-focus stimuli (*z* = 4.49, *p*<0.0001). Furthermore, they achieved better discrimination when the two stimuli in a pair had greater numbers of different tones (*z* = 2.27, *p* = 0.02).

**Figure 4:**
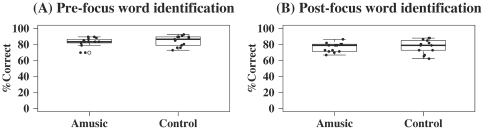
Boxplots of amusics and controls' scores on the two word identification tasks (in percentage of correct responses; %Correct). (A) pre-focus word identification, and (B) post-focus word identification.

Figure 4 shows the results on the word identification tasks (see Tables S4 and S5 for individual scores and reaction times). Mixed-effects ANOVA with Subject (individual participants) as the random effect, Group (amusic versus control) the between-subject factor, and Focus (pre versus post) the within-subject factor revealed a significant effect of Focus [F(1,24) = 35.66, *p*<0.0001]. Neither Group [F(1,24) = 0.37, *p* = 0.55] nor Group×Focus interaction [F(1,24) = 0.05, *p* = 0.83] was significant. This indicates that amusics performed as well as controls on both pre- and post-focus word identification. Both groups performed significantly better on pre- than post-focus word identification [amusics: F(1,12) = 16.80, *p* = 0.001; controls: F(1,12) = 18.88, *p* = 0.001]. Both groups' performances on the two tasks were positively correlated (amusics: *z* = 2.27, *p* = 0.02, *τ* = 0.51; controls: *z* = 2.66, *p* = 0.008, *τ* = 0.58).

### Statement-question discrimination and identification

**Figure 5:**
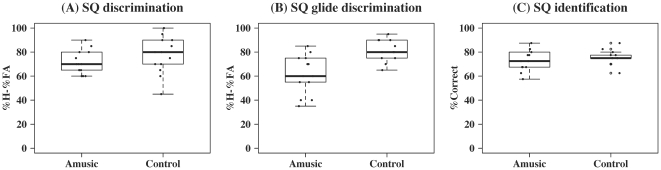
Boxplots of amusics and controls' scores on the three statement-question perception tasks. (A) statement-question discrimination in natural speech (in %H – %FA), (B) statement-question discrimination in gliding tones (in %H – %FA), and (C) statement-question identification in natural speech (in %Correct).

Figure 5 shows the results on the intonation tasks (see Tables S6 and S7 for individual scores and reaction times). No significant group difference was observed for the identification task [F(1,24) = 0.73, *p* = 0.40]. For the discrimination tasks, mixed-effects ANOVA with Subject (individual participants) as the random effect, Group (amusic versus control) the between-subject factor, and Stimulus (natural speech versus gliding tone) the within-subject factor revealed significant effects of Group [F(1,24) = 9.76, *p* = 0.005] and Group×Stimulus interaction [F(1,24) = 5.19, *p* = 0.03], but not Stimulus [F(1,24) = 1.03, *p* = 0.32]. This was because amusics achieved normal performance on natural speech [F(1,24) = 1.30, *p* = 0.27] but showed impaired performance on gliding tone analogs [F(1,24) = 14.91, *p* = 0.0007]. Furthermore, while controls' performances did not differ significantly across the two stimulus types [F(1,12) = 0.56, *p* = 0.47], amusics performed significantly better on natural speech than on gliding tone analogs [F(1,12) = 9.45, *p* = 0.0096]. Interestingly, while amusics' performances on the two discrimination tasks showed a significant positive correlation (*z* = 2.38, *p* = 0.02, *τ* = 0.53), controls' performances on the two tasks were not significantly correlated (*z* = −0.57, *p* = 0.57, *τ* = −0.13).

Analysis of the errors made by amusics in the two discrimination tasks (natural speech and gliding tones) indicates that there were more misses than false alarms (83.8% versus 16.2% in natural speech; 90% versus 10% in gliding tones). Given that natural speech stimuli and their gliding tone analogs shared the same pitch and duration patterns, but differed slightly in intensity envelopes, two separate generalized linear mixed models were fit to examine what might have caused amusics' insensitivity to ‘different’ pairs in the two discrimination tasks, with stimulus presentation order (statement-question versus question-statement), sentence length (3–7 syllables), tone component (High versus Falling), focus condition (initial versus final), and the absolute differences in acoustic characteristics between the two stimuli in a pair (see Table 3) as fixed effects, and individual participants and stimulus items as random effects. Results indicate that in the model for amusics' responses to ‘different’ speech stimuli, only the absolute difference in overall mean intensity between the stimuli in a pair significantly affected amusics' performance, although in an unexpected direction: the bigger the absolute difference, the worse the performance (*z* = −2.15, *p* = 0.03). On the other hand, amusics' discrimination performance on gliding tone analogs was significantly affected by several acoustic characteristics of the stimulus pairs. Among them, most effects were in expected directions, namely, amusics performed significantly better on gliding tone pairs that differed greatly in overall mean F_0_ (*z* = 2.78, *p* = 0.005), overall pitch range (*z* = 2.04, *p* = 0.04), final glide time (*z* = 3.09, *p* = 0.002), final syllable duration (*z* = 2.03, *p* = 0.04), and final mean intensity (*z* = 2.26, *p* = 0.02). Nevertheless, two effects worked in unexpected directions, with amusics showing better discrimination performance on gliding tone pairs that had smaller differences in overall duration (*z* = −2.68, *p* = 0.007) and final glide rate (*z* = −2.05, *p* = 0.04). Interestingly, none of the acoustic effects or other fixed effects of the stimulus characteristics contributed significantly to controls' discrimination of statements and questions and their gliding tone analogs.

### Pitch threshold tasks

**Figure 6:**
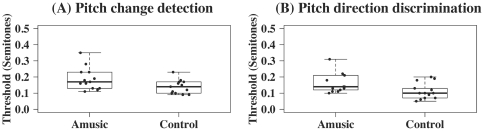
Boxplots of amusics and controls' pitch thresholds (in st) in the two psychophysical tasks. (A) pitch change detection, and (B) pitch direction discrimination.

Figure 6 shows the results on pitch threshold tasks (see Table S6 for individual scores). Mixed-effects ANOVA with Subject (individual participants) as the random effect, Group (amusic versus control) the between-subject factor, and Task (pitch change detection versus pitch direction discrimination) the within-subject factor revealed significant effects of Group [F(1,24) = 6.21, *p* = 0.02] and Task [F(1,24) = 6.78, *p* = 0.02], but not Group×Task interaction [F(1,24) = 0.01, *p* = 0.94]. That is, amusics had significantly higher pitch thresholds than controls for both pitch change detection and pitch direction discrimination. Both groups showed a tendency to perform better on pitch direction discrimination than pitch change detection. No significant correlation was found for either group between their performances on the two pitch threshold tasks.

Correlation analyses between word/intonation tasks and pitch threshold tasks indicate that amusics' performance on post-focus word identification was negatively correlated with their thresholds for both pitch change detection (*z* = −2.17, *p* = 0.03, *τ* = −0.48) and pitch direction discrimination (*z* = −2.05, *p* = 0.04, *τ* = −0.45). Controls' performance on statement-question identification was negatively correlated with their thresholds for pitch direction discrimination (*z* = −2.22, *p* = 0.03, *τ* = −0.50). That is, the smaller the pitch thresholds, the better the performance on those speech tasks.

## Discussion

### Speech processing in Mandarin amusics

Although previous studies have suggested that amusia impacts upon speech processing in subtle ways for speakers of both tone and non-tonal languages [4]–[8], it was unclear whether the ‘lexical tone agnosia’ reported for Mandarin amusics was caused by pitch-processing deficits or impaired phonological awareness [7]. This study investigated the mechanism of speech processing in Mandarin amusics by employing different experimental designs than previous studies.

First, by using relatively small tonal contrasts in word discrimination that involved the same segments and by providing Chinese characters in word identification, we found impaired performance on word discrimination but normal performance on word identification in our Mandarin amusics. This is in contrast to what was observed for the subgroup of Mandarin amusics with ‘lexical tone agnosia’ in [7]. The conflicting results on word/tone discrimination between the current study and [7] are likely due to the fact that our stimuli contained much smaller pitch excursion sizes than those in [7] (1.5–4.1 st versus 2–17 st), making it possible to reveal amusics' pitch-processing deficits in linguistic tone processing even when the tones shared the same segments. The discrepancy regarding tone/word identification between [7] and the current study is likely due to the different demands for phonological awareness between the two tasks. While the tone identification task in [7] required explicit labeling of tone names (thus demanding a high level of phonological awareness), our task required recognition of the Chinese characters that represented the words with the tones.

Second, by using naturally spoken statements and questions that differed in multiple acoustic cues across the entire utterances, we found normal performance on statement-question discrimination and identification in our Mandarin amusics. This is in contrast to the findings in [5] where Mandarin amusics showed subtle problems with identification of statements and questions that differed mainly in final pitch. This indicates that human listeners including amusics are adept at using multiple acoustic cues (F_0_, duration, and intensity) to achieve speech communication.

However, it is puzzling that amusics showed inferior performance on word discrimination but normal performance on word identification with exactly the same set of stimuli in the current study. This is unlikely due to the order in which word discrimination and identification were presented, since the results were robust across pre- and post-focus conditions and across groups. Moreover, both groups demonstrated increased response latencies for word identification compared with discrimination in terms of reaction times (Table S5). According to [42], short-term memory of two auditory events is required in discrimination tasks, whereas the comparison between the long-term memory store and a single auditory event is needed in identification tasks. Given that amusics have short-term memory deficits for pitch [43]–[44] but no obvious long-term memory impairment [24], it is possible that controls' superior word/glide discrimination performance can be accounted for by their enhanced short-term memory for pitch relative to amusics. However, the analysis of amusics' responses to ‘different’ word pairs did not find a significant main effect of stimulus length (words ranging from 1 to 4 syllables). Rather, the errors were mainly caused by amusics' failure to detect the small pitch differences between the two words in a pair. Mandarin speakers have been shown to be able to identify the four lexical tones correctly 90% of the time with a pitch range only around 0.49 st, and they could identify Tones 1 and 4 efficiently even at the pitch range of 0.25 st [45]. Since the pitch ranges of our tone stimuli were around 1.5–4.1 st (Table S1), they did not seem to be small enough to jeopardize amusics' word identification performance. This is reminiscent of the previous finding that listeners can process linguistic contrasts based on acoustic differences they cannot consciously recognize [46]–[47].

### Pitch thresholds in Mandarin speakers

It is a matter of debate whether psychophysical pitch discrimination is a basic low-level ability or is shaped by linguistic/musical experience [48]–[49]. Previous studies have shown that amusics have significantly higher thresholds than controls for both pitch change detection and pitch direction discrimination, but the difference in pitch direction discrimination is especially pronounced between the two groups [6], [22]. In the current study, although our Mandarin amusics also demonstrated higher pitch thresholds than controls for pitch change detection and pitch direction discrimination, both groups exhibited slightly better (smaller) thresholds for pitch direction discrimination than pitch change detection. Furthermore, although highly comparable on the MBEA scores (all *p*s>0.1), the Mandarin groups in the current study performed significantly better than the English groups in [6] on pitch direction discrimination (Wilcoxon rank sum test: English versus Mandarin control groups: *W* = 42, *p* = 0.04; amusic groups: *W* = 16, *p*<0.0001), but not on pitch change detection (control groups: *W* = 72.5, *p* = 0.69; amusic groups: *W* = 82, *p* = 0.08).

It has been shown that the pitch direction thresholds of typical individuals are considerably higher than their pitch change thresholds [45] (although see [50] for mixed results). The remarkably lower thresholds for pitch direction discrimination in both amusic and normal Mandarin speakers in the current study may reflect ‘perceptual learning’ (e.g., [51]–[52]) or ‘experience-dependent plasticity’ (e.g., [53]). In Mandarin, tones such as Rising and Falling are the fundamental ‘building blocks’ of everyday speech. In English, however, only focused or sentence-final stressed syllables carry deliberate pitch changes [9], [11]. As a consequence, Mandarin speech contains more dynamic F_0_ movements, and is characterized by greater rates of F_0_ changes than English speech [54]. Multidimensional scaling studies on tone perception have demonstrated that linguistic experience shapes listeners' perceptual dimensions of tone [55]–[57]. For example, Mandarin listeners attached more importance to the ‘direction’ dimension (rising versus non-rising) than the ‘height’ dimension (average F_0_ level) in their judgments of tone dissimilarity, while English listeners showed the opposite pattern. This is again in line with the ‘perceptual learning’ theory [52: 592–594], according to which individuals may develop specialized ‘feature detectors’ or ‘internal representations’ for perceived stimuli through ‘feature imprinting’ of ‘environmental inputs’. Indeed, there is evidence for ‘experience-dependent plasticity’ in tone language speakers and musicians (e.g., [53]). For example, Mandarin speakers and English non-musicians and musicians exhibited ‘enhanced tuning’ only to the pitch features that are most relevant to their native language (‘direction’ or ‘pitch acceleration’ in Mandarin versus ‘height’ in English) and to music (‘musical pitch interval’) during pre-attentive pitch processing in the auditory brainstem [53: 432]. This may in part explain why Mandarin amusics still suffer from amusia despite exhibiting relatively small pitch direction discrimination thresholds: tuning to different pitch features is required in linguistic versus musical processing. It will be interesting to examine Mandarin amusics' frequency-following responses to linguistic tones and musical intervals in the brainstem, in comparison to normal controls and musicians, as the results are likely to provide insight into why amusia only affects speech processing in subtle ways.

### The effect of stimulus type on pitch processing

As in previous studies [2], [5]–[6], [8], [10], the results on the effect of stimulus type on pitch processing are also mixed in the current study. In the word/glide discrimination tasks, amusics achieved better performance on gliding tones than on natural words. However, they performed significantly worse on gliding tones than on natural speech in the statement-question discrimination tasks. Given that our word stimuli ranged from one to four syllables and our sentence stimuli from three to seven syllables, it is possible that amusics' inferior performance on discrimination of the gliding tone analogs of statements and questions was caused by their short-term memory deficits for tones [43]–[44]. On the other hand, both English and Cantonese listeners showed higher sensitivity to F_0_ differences for non-speech complex tones than synthesized speech stimuli [58]. Since our gliding tone analogs were also made of complex tones, they should not bring any disadvantage to the listeners in pitch processing as compared to speech sounds. However, there are other substantial differences between speech materials and tone analogs, e.g., the presence/absence of linguistic information, which might have led to the different performance in amusics [1], [10]. A more matched comparison between speech and music processing in amusia could adopt the approach in [59]–[60], comparing speaking versus singing performance in amusics.

Overall, the findings of the current study suggest that the mechanism of speech processing in amusia is unlikely to be different across tone and non-tonal language speakers. Rather, the disorder appears to be a domain-general pitch-processing deficit that is neither music-specific nor language-specific. Nevertheless, in everyday life, it only manifests itself in the musical domain, and it is only under laboratory conditions that tone/intonation processing deficits in speech can be revealed. However, given the relatively small sample size of the current study and the heterogeneity of the amusic population [2]–[8], [22], [24]–[25], future studies on a larger sample of Mandarin amusics and on amusics of other language backgrounds are needed to further corroborate the current findings.

## Supporting Information

Table S1 Glide size/time/rate of the tones in post- versus pre-focus words.
(DOC)

Table S2 Characteristics of the participants.
(DOC)

Table S3 A set of statement-question pairs used in intonation tasks.
(DOC)

Table S4 Performance of amusics (A1-13) and controls (C1-13) on word perception tasks.
(DOC)

Table S5 Percentages of correct and incorrect responses and reaction times on word perception tasks by amusics and controls.
(DOC)

Table S6 Performance of amusics (A1-13) and controls (C1-13) on pitch threshold and intonation tasks.
(DOC)

Table S7 Percentages of correct and incorrect responses and reaction times on intonation perception tasks by amusics and controls.
(DOC)
