# Supplementary material for: The Mechanism of Speech Processing in Congenital Amusia: Evidence from Mandarin Speakers
Source: PLoS One. 2012 Feb 8;7(2):e30374. doi: 10.1371/journal.pone.0030374 (PMC3275596; doi:10.1371/journal.pone.0030374)
Supplement: Table S1 — Glide size/time/rate of the tones in post- versus pre-focus words. (DOC) [file pone.0030374.s001.doc]

**Table S1.** Glide size/time/rate of the tones in post- versus pre-focus words. Note: Tone 1 = High; Tone 2 = Rising; Tone 3 = Low; Tone 4 = Falling; *μ* = mean; *σ* = standard deviation; *t* is the statistic of the paired *t*-test (two-tailed); glide size (in st) is the pitch excursion size of the tone (= maximum F_0_ – minimum F_0_); glide time (in s) is the duration between maximum and minimum F_0_ of the tone; glide rate (in st/s) = glide size / glide time.

| Focus |  | Tone 1 (*n* = 47) | | | Tone 2 (*n* = 51) | | | Tone 3 (*n* = 9) | | | Tone 4 (*n* = 55) | | |
| --- | --- | --- | --- | --- | --- | --- | --- | --- | --- | --- | --- | --- | --- |
|  |  | Glide  Size | Glide  Time | Glide  Rate | Glide  Size | Glide  Time | Glide  Rate | Glide  Size | Glide  Time | Glide  Rate | Glide  Size | Glide  Time | Glide  Rate |
| Post-  focus | *μ* | 1.51 | 0.08 | -7.82 | 1.79 | 0.07 | -17.92 | 1.72 | 0.07 | -23.68 | 2.03 | 0.09 | -19.04 |
|  | *σ* | 1.15 | 0.04 | 29.81 | 1.17 | 0.03 | 24.84 | 1.19 | 0.02 | 14.79 | 1.32 | 0.03 | 20.19 |
| Pre-  focus | *μ* | 1.50 | 0.09 | -1.56 | 1.73 | 0.07 | -13.86 | 4.11 | 0.06 | -22.09 | 2.27 | 0.10 | -24.77 |
|  | *σ* | 0.98 | 0.04 | 38.31 | 1.09 | 0.03 | 30.80 | 3.99 | 0.03 | 68.93 | 1.56 | 0.04 | 18.08 |
| *t*-test | *t* | 0.05 | -0.90 | -0.90 | 0.38 | 0.86 | -0.71 | -2.03 | 0.56 | -0.07 | -1.03 | -2.28 | 1.56 |
|  | *df* | 46 | 46 | 46 | 50 | 50 | 50 | 8 | 8 | 8 | 54 | 54 | 54 |
|  | *P* | 0.96 | 0.37 | 0.37 | 0.70 | 0.39 | 0.48 | 0.08 | 0.59 | 0.95 | 0.31 | 0.03 | 0.12 |
