# Supplementary material for: The Mechanism of Speech Processing in Congenital Amusia: Evidence from Mandarin Speakers
Source: PLoS One. 2012 Feb 8;7(2):e30374. doi: 10.1371/journal.pone.0030374 (PMC3275596; doi:10.1371/journal.pone.0030374)
Supplement: Table S2 — Characteristics of the participants. (DOC) [file pone.0030374.s002.doc]

**Table S2.** Characteristics of the participants. The MBEA (Montreal Battery of Evaluation of Amusia; Peretz et al., 2003) subset scores are in number of correct responses out of 30. Note: A1-A13: amusics; C1-C13: controls; F = female; M = male; L = left; R = right; Pitch composite = scale + contour + interval. None of the participants received formal extra-curricular musical training. However, C6 took music lessons at school for 1.5 years, and C13 learned guitar by herself for 2 years.

| Participant | Sex | Handed-  ness | Age | Education | Scale | Contour | Interval | Rhythm | Meter | Memory | Pitch  composite |
| --- | --- | --- | --- | --- | --- | --- | --- | --- | --- | --- | --- |
| A1 | M | R | 26 | 19 | 16 | 19 | 17 | 26 | 15 | 15 | 52 |
| A2 | F | R | 26 | 20 | 12 | 16 | 15 | 15 | 19 | 19 | 43 |
| A3 | M | L | 27 | 19 | 18 | 17 | 21 | 27 | 23 | 21 | 56 |
| A4 | M | R | 21 | 15 | 13 | 17 | 14 | 17 | 11 | 21 | 44 |
| A5 | F | R | 22 | 15 | 19 | 18 | 21 | 26 | 21 | 29 | 58 |
| A6 | M | R | 25 | 18 | 22 | 19 | 18 | 16 | 25 | 27 | 59 |
| A7 | F | R | 24 | 16 | 14 | 16 | 15 | 15 | 17 | 17 | 45 |
| A8 | F | L | 23 | 17 | 19 | 20 | 21 | 24 | 22 | 28 | 60 |
| A9 | F | R | 22 | 14 | 16 | 20 | 17 | 21 | 20 | 16 | 53 |
| A10 | F | R | 21 | 12 | 19 | 20 | 21 | 25 | 25 | 22 | 60 |
| A11 | F | R | 24 | 17 | 20 | 19 | 23 | 24 | 19 | 22 | 62 |
| A12 | F | R | 21 | 14 | 12 | 25 | 22 | 25 | 16 | 24 | 59 |
| A13 | M | R | 31 | 20 | 20 | 25 | 18 | 24 | 21 | 19 | 63 |
| C1 | M | R | 24 | 18 | 30 | 29 | 28 | 29 | 28 | 30 | 87 |
| C2 | F | R | 24 | 19 | 25 | 28 | 29 | 25 | 25 | 28 | 82 |
| C3 | M | R | 24 | 16 | 26 | 27 | 24 | 25 | 25 | 22 | 77 |
| C4 | F | R | 26 | 19 | 27 | 25 | 26 | 24 | 26 | 28 | 78 |
| C5 | M | R | 23 | 17 | 28 | 26 | 27 | 27 | 27 | 28 | 81 |
| C6 | M | R | 25 | 18 | 23 | 27 | 27 | 26 | 25 | 29 | 77 |
| C7 | F | R | 21 | 17 | 28 | 24 | 26 | 27 | 26 | 29 | 78 |
| C8 | F | R | 26 | 18 | 27 | 25 | 26 | 27 | 22 | 29 | 78 |
| C9 | F | R | 25 | 17 | 26 | 28 | 26 | 29 | 28 | 29 | 80 |
| C10 | F | R | 25 | 18 | 26 | 29 | 28 | 29 | 30 | 28 | 83 |
| C11 | F | R | 25 | 19 | 27 | 25 | 23 | 29 | 29 | 28 | 75 |
| C12 | F | R | 24 | 19 | 28 | 27 | 26 | 27 | 23 | 30 | 81 |
| C13 | F | R | 29 | 18 | 30 | 29 | 27 | 28 | 28 | 29 | 86 |
