# Supplementary material for: The Mechanism of Speech Processing in Congenital Amusia: Evidence from Mandarin Speakers
Source: PLoS One. 2012 Feb 8;7(2):e30374. doi: 10.1371/journal.pone.0030374 (PMC3275596; doi:10.1371/journal.pone.0030374)
Supplement: Table S3 — A set of statement-question pairs used in intonation tasks. (DOC) [file pone.0030374.s003.doc]

**Table S3.** A set of statement-question pairs used in intonation tasks. These utterances were naturally spoken with either an initial or a final focus that was elicited by prompt sentences during recording (note that these prompts were not presented during testing). In the table, the sentences in Column 2 were prompts, and those in Column 3 were target sentences (note that the word 那 ‘then’ was not included in the question stimuli). Therefore, these statements and questions shared the same word sequence but differed in intonation.

| Sentence type | Prompt sentences | Target sentences |
| --- | --- | --- |
| Statement with  initial focus | **谁**做饭？  ‘**Who** cooks the rice?’ | **顾俊**做饭。  ‘**GuJun** cooks the rice.’ |
| Question with  initial focus | 不是**赵亮**做饭。  ‘It is not **ZhangLiang** who cooks the rice.’ | [那]**顾俊**做饭？  ‘[Then] **GuJun** cooks the rice?’ |
| Statement with  final focus | 顾俊**干什么**？  ‘**What** does GuJun do?’ | 顾俊**做饭**。  ‘GuJun **cooks the rice**.’ |
| Question with  final focus | 顾俊不**炒菜**。  ‘GuJun does not **stir-fry the dish**.’ | [那]顾俊**做饭**？  ‘[Then] GuJun **cooks the rice**?’ |
