# Supplementary material for: The Mechanism of Speech Processing in Congenital Amusia: Evidence from Mandarin Speakers
Source: PLoS One. 2012 Feb 8;7(2):e30374. doi: 10.1371/journal.pone.0030374 (PMC3275596; doi:10.1371/journal.pone.0030374)
Supplement: Table S4 — Performance of amusics (A1-13) and controls (C1-13) on word perception tasks. (DOC) [file pone.0030374.s004.doc]

**Table S4.** Performance of amusics (A1-13) and controls (C1-13) on word perception tasks. Note: %H-%FA = percentage of hits – percentage of false alarms; %Correct = percentage of correct responses.

| Participant | Pre-focus word discrimination  (%H-%FA) | | Post-focus word discrimination  (%H-%FA) | | Pre-focus word identification  (%Correct) | Post-focus word identification  (%Correct) |
| --- | --- | --- | --- | --- | --- | --- |
|  | Natural speech | Gliding tones | Natural speech | Gliding tones |  |  |
| A1 | 72.7 | 84.8 | 54.5 | 66.7 | 83.3 | 78.8 |
| A2 | 42.4 | 57.6 | 54.5 | 51.5 | 83.3 | 80.3 |
| A3 | 84.8 | 87.9 | 81.8 | 75.8 | 81.8 | 69.7 |
| A4 | 60.6 | 78.8 | 54.5 | 78.8 | 87.9 | 78.8 |
| A5 | 81.8 | 81.8 | 69.7 | 78.8 | 84.8 | 78.8 |
| A6 | 75.8 | 93.9 | 69.7 | 75.8 | 83.3 | 81.8 |
| A7 | 57.6 | 33.3 | 54.5 | 66.7 | 83.3 | 71.2 |
| A8 | 75.8 | 97.0 | 72.7 | 93.9 | 89.4 | 80.3 |
| A9 | 48.5 | 81.8 | 54.5 | 81.8 | 69.7 | 72.7 |
| A10 | 69.7 | 90.9 | 63.6 | 87.9 | 78.8 | 66.7 |
| A11 | 57.6 | 81.8 | 51.5 | 78.8 | 89.4 | 86.4 |
| A12 | 63.6 | 87.9 | 57.6 | 63.6 | 69.7 | 71.2 |
| A13 | 60.6 | 81.8 | 48.5 | 87.9 | 86.4 | 72.7 |
| Mean | 65.5 | 79.9 | 60.6 | 76.0 | 82.4 | 76.1 |
| SD | 12.6 | 16.9 | 10.0 | 11.5 | 6.4 | 5.7 |
| C1 | 90.9 | 97.0 | 69.7 | 90.9 | 92.4 | 87.9 |
| C2 | 87.9 | 100.0 | 66.7 | 78.8 | 89.4 | 87.9 |
| C3 | 90.9 | 100.0 | 90.9 | 87.9 | 89.4 | 72.7 |
| C4 | 84.8 | 93.9 | 75.8 | 90.9 | 90.9 | 80.3 |
| C5 | 78.8 | 93.9 | 69.7 | 81.8 | 86.4 | 72.7 |
| C6 | 78.8 | 78.8 | 69.7 | 84.8 | 80.3 | 78.8 |
| C7 | 60.6 | 84.8 | 63.6 | 81.8 | 75.8 | 62.1 |
| C8 | 81.8 | 100.0 | 78.8 | 87.9 | 89.4 | 84.8 |
| C9 | 90.9 | 90.9 | 60.6 | 87.9 | 87.9 | 86.4 |
| C10 | 66.7 | 87.9 | 66.7 | 97.0 | 78.8 | 78.8 |
| C11 | 66.7 | 97.0 | 75.8 | 84.8 | 72.7 | 66.7 |
| C12 | 75.8 | 93.9 | 60.6 | 69.7 | 75.8 | 65.2 |
| C13 | 81.8 | 97.0 | 69.7 | 84.8 | 84.8 | 81.8 |
| Mean | 79.7 | 93.5 | 70.6 | 85.3 | 84.2 | 77.4 |
| SD | 10.0 | 6.4 | 8.2 | 6.7 | 6.6 | 8.8 |
