# Supplementary material for: The Mechanism of Speech Processing in Congenital Amusia: Evidence from Mandarin Speakers
Source: PLoS One. 2012 Feb 8;7(2):e30374. doi: 10.1371/journal.pone.0030374 (PMC3275596; doi:10.1371/journal.pone.0030374)
Supplement: Table S5 — Percentages of correct and incorrect responses and reaction times on word perception tasks by amusics and controls. (DOC) [file pone.0030374.s005.doc]

**Table S5.** Percentages of correct and incorrect responses and reaction times on word perception tasks by amusics and controls. Note: %Correct: percentage of correct responses; %Incorrect: percentage of incorrect responses; Task 1: pre-focus word discrimination; Task 2: pre-focus glide discrimination; Task 3: pre-focus word identification; Task 4: post-focus word discrimination; Task 5: post-focus glide discrimination; Task 6: post-focus word identification.

| Measure | | %Correct | | | %Incorrect | | |
| --- | --- | --- | --- | --- | --- | --- | --- |
|  |  | Amusics | Controls | Wilcoxon rank sum test | Amusics | Controls | Wilcoxon rank sum test |
|  |  | Mean (SD) | Mean (SD) | *W* (*P*) | Mean (SD) | Mean (SD) | *W* (*P*) |
| Accuracy | Task 1 | 82.8 (6.3) | 89.9 (5.0) | 30.5 (0.005) | 17.2 (6.3) | 10.1 (5.0) | 138.5 (0.005) |
|  | Task 2 | 90.0 (8.5) | 96.7 (3.2) | 28.5 (0.004) | 10.0 (8.5) | 3.3 (3.2) | 140.5 (0.004) |
|  | Task 3 | 82.4 (6.4) | 84.1 (6.7) | 67 (0.37) | 17.6 (6.4) | 15.9 (6.7) | 102 (0.37) |
|  | Task 4 | 80.3 (5.0) | 85.3 (4.1) | 37.5 (0.01) | 19.7 (5.0) | 14.7 (4.1) | 131.5 (0.01) |
|  | Task 5 | 88.0 (5.8) | 92.7 (3.3) | 38.5 (0.02) | 12.0 (5.8) | 7.3 (3.3) | 130.5 (0.02) |
|  | Task 6 | 76.1 (5.7) | 77.4 (8.8) | 71.5 (0.50) | 23.9 (5.7) | 22.6 (8.8) | 97.5 (0.50) |
| Reaction time | Task 1 | 764.4  (356.9) | 689.9  (295.3) | 91  (0.76) | 1220.6 (806.3) | 1024.9 (550.4) | 90  (0.80) |
|  | Task 2 | 542.6  (139.9) | 551.4  (176.6) | 87  (0.92) | 871.5 (296.1) | 1000.6 (622.3) | 65  (1) |
|  | Task 3 | 1236.1  (426.4) | 1191.1 (343.2) | 84  (1) | 1580.2 (792.4) | 1912.9 (906.8) | 62  (0.26) |
|  | Task 4 | 726.1  (232.0) | 713.5  (160.1) | 85  (1) | 1099.8 (518.9) | 1048.7 (459.2) | 84  (1) |
|  | Task 5 | 620.9  (168.2) | 614.4  (174.6) | 88  (0.88) | 917.1 (469.1) | 913.1 (408.2) | 83  (0.96) |
|  | Task 6 | 1136.3  (375.8) | 1297.3 (521.2) | 68  (0.42) | 1397.8 (591.0) | 1463.0 (686.2) | 79  (0.80) |
