# Supplementary material for: The Mechanism of Speech Processing in Congenital Amusia: Evidence from Mandarin Speakers
Source: PLoS One. 2012 Feb 8;7(2):e30374. doi: 10.1371/journal.pone.0030374 (PMC3275596; doi:10.1371/journal.pone.0030374)
Supplement: Table S6 — Performance of amusics (A1-13) and controls (C1-13) on pitch threshold and intonation tasks. (DOC) [file pone.0030374.s006.doc]

**Table S6.** Performance of amusics (A1-13) and controls (C1-13) on pitch threshold and intonation tasks. Note: %H-%FA = percentage of hits – percentage of false alarms; %Correct = percentage of correct responses; st = semitone.

| Participant | Pitch threshold  (st) | | Statement-question  discrimination (%H-%FA) | | Statement-question  identification  (%Correct) |
| --- | --- | --- | --- | --- | --- |
|  | Pitch change detection | Pitch direction  discrimination | Natural speech | Gliding tones |  |
| A1 | 0.19 | 0.21 | 60.0 | 35.0 | 72.5 |
| A2 | 0.13 | 0.11 | 60.0 | 40.0 | 62.5 |
| A3 | 0.16 | 0.22 | 80.0 | 75.0 | 67.5 |
| A4 | 0.23 | 0.13 | 65.0 | 40.0 | 77.5 |
| A5 | 0.11 | 0.13 | 80.0 | 80.0 | 72.5 |
| A6 | 0.13 | 0.10 | 85.0 | 70.0 | 67.5 |
| A7 | 0.35 | 0.31 | 65.0 | 75.0 | 57.5 |
| A8 | 0.18 | 0.12 | 60.0 | 70.0 | 67.5 |
| A9 | 0.16 | 0.21 | 70.0 | 55.0 | 82.5 |
| A10 | 0.28 | 0.18 | 90.0 | 85.0 | 82.5 |
| A11 | 0.12 | 0.14 | 75.0 | 55.0 | 77.5 |
| A12 | 0.23 | 0.11 | 70.0 | 60.0 | 80.0 |
| A13 | 0.17 | 0.14 | 70.0 | 60.0 | 87.5 |
| Mean | 0.19 | 0.16 | 71.5 | 61.5 | 73.5 |
| SD | 0.07 | 0.06 | 9.9 | 16.1 | 8.8 |
| C1 | 0.09 | 0.09 | 75.0 | 85.0 | 75.0 |
| C2 | 0.14 | 0.05 | 85.0 | 75.0 | 80.0 |
| C3 | 0.17 | 0.12 | 60.0 | 80.0 | 75.0 |
| C4 | 0.12 | 0.10 | 80.0 | 95.0 | 87.5 |
| C5 | 0.17 | 0.06 | 70.0 | 65.0 | 82.5 |
| C6 | 0.18 | 0.19 | 100.0 | 80.0 | 75.0 |
| C7 | 0.10 | 0.10 | 80.0 | 80.0 | 70.0 |
| C8 | 0.15 | 0.20 | 65.0 | 90.0 | 62.5 |
| C9 | 0.23 | 0.18 | 95.0 | 75.0 | 75.0 |
| C10 | 0.11 | 0.07 | 70.0 | 90.0 | 77.5 |
| C11 | 0.10 | 0.07 | 90.0 | 90.0 | 75.0 |
| C12 | 0.16 | 0.13 | 45.0 | 80.0 | 75.0 |
| C13 | 0.09 | 0.10 | 90.0 | 70.0 | 77.5 |
| Mean | 0.14 | 0.11 | 77.3 | 81.2 | 76.0 |
| SD | 0.04 | 0.05 | 15.4 | 8.7 | 5.9 |
