# Supplementary material for: The Mechanism of Speech Processing in Congenital Amusia: Evidence from Mandarin Speakers
Source: PLoS One. 2012 Feb 8;7(2):e30374. doi: 10.1371/journal.pone.0030374 (PMC3275596; doi:10.1371/journal.pone.0030374)
Supplement: Table S7 — Percentages of correct and incorrect responses and reaction times on intonation perception tasks by amusics and controls. (DOC) [file pone.0030374.s007.doc]

**Table S7.** Percentages of correct and incorrect responses and reaction times on intonation perception tasks by amusics and controls. Note: %Correct: percentage of correct responses; %Incorrect: percentage of incorrect responses; Task 1: statement-question discrimination in natural speech; Task 2: statement-question discrimination in gliding tones; Task 3: statement-question identification in natural speech.

| Measure | | %Correct | | | %Incorrect | | |
| --- | --- | --- | --- | --- | --- | --- | --- |
|  |  | Amusics | Controls | Wilcoxon rank sum test | Amusics | Controls | Wilcoxon rank sum test |
|  |  | Mean (SD) | Mean (SD) | *W* (*P*) | Mean (SD) | Mean (SD) | *W* (*P*) |
| Accuracy | Task 1 | 85.8 (4.9) | 88.7 (7.7) | 59.5 (0.20) | 14.2 (4.9) | 11.3 (7.7) | 109.5 (0.20) |
|  | Task 2 | 80.8 (8.1) | 90.6 (4.3) | 23.5 (0.002) | 19.2 (8.1) | 9.4 (4.3) | 145.5 (0.002) |
|  | Task 3 | 73.5 (8.8) | 76.0 (5.9) | 71.5 (0.50) | 26.5 (8.8) | 24.0 (5.9) | 97.5 (0.50) |
| Reaction time | Task 1 | 531.1  (174.8) | 674.4  (282.0) | 63  (0.29) | 806.1 (399.6) | 1390.2 (897.0) | 50  (0.14) |
|  | Task 2 | 401.4  (160.4) | 438.0  (130.0) | 63  (0.29) | 695.0 (416.7) | 700.6 (465.4) | 90  (0.80) |
|  | Task 3 | 696.2  (233.5) | 867.3  (442.2) | 63  (0.29) | 937.4 (492.8) | 1165.1 (517.0) | 56  (0.15) |
